# Prevalence of Lynch syndrome in women with mismatch repair‐deficient ovarian cancer

**DOI:** 10.1002/cam4.3688

**Published:** 2020-12-25

**Authors:** Rachel Hodan, Kerry Kingham, Kristina Cotter, Ann K. Folkins, Allison W. Kurian, James M. Ford, Teri Longacre

**Affiliations:** ^1^ Cancer Genetics and Genomics Stanford Health Care Stanford CA USA; ^2^ Department of Pediatrics (Genetics) Stanford University School of Medicine Stanford CA USA; ^3^ Department of Pathology Stanford University School of Medicine Stanford CA USA; ^4^ Department of Oncology Stanford University School of Medicine Stanford CA USA; ^5^ Department of Epidemiology and Population Health Stanford University School of Medicine Stanford CA USA; ^6^ Department of Genetics Stanford University School of Medicine Stanford CA USA

**Keywords:** germline, Lynch syndrome, mismatch repair, ovarian cancer, universal tumor screening

## Abstract

**Background:**

There are limited data on the prevalence of Lynch syndrome (LS) in women with primary ovarian cancer with mismatch repair deficiency (MMR‐D) by immunohistochemistry (IHC).

**Materials and Methods:**

Three hundred and eight cases of primary ovarian, fallopian, and peritoneal cancer between January 2012 and December 2019 were evaluated for MMR‐D by IHC. The incidence of LS in this cohort was evaluated.

**Results:**

MMR‐D by IHC was identified in 16 of 308 (5.2%) (95% CI: 3.2%–8.3%) primary ovarian‐related cancers. Most cases with MMR‐D were endometrioid (*n* = 11, 68.7%); (95% CI: 44.2%–86.1%). MSH2/MSH6 protein loss was detected in eight cases (50.0%); (95% CI: 28.0%–72.0%) and MLH1/PMS2 protein loss was detected in four cases (25.0%); (95% CI: 9.7%–50.0%). MSH6 protein loss was detected in two cases (12.5%); (95% CI: 2.2%–37.3%) and PMS2 protein loss was detected in two cases (12.5%); (95% CI: 2.2%–37.3%). All four cases with MLH1/PMS2 protein loss had *MLH1* promotor hypermethylation. All 12 women with ovarian cancer suggestive of LS underwent germline testing and 8 (66.6%); (95% CI: 38.8%–86.5%) were confirmed to have LS.

**Conclusions:**

Most ovarian cancers with somatic MMR‐D were confirmed to have LS in this cohort. Germline testing for LS in addition to *BRCA1*/*2* for all women with an epithelial ovarian cancer would be efficient and would approach 100% sensitivity for identifying Lynch syndrome. Utilization of a multigene panel should also be considered, given the additional non‐Lynch germline mutation identified in this cohort.

## Graphical abstract

The majority of mismatch repair deficient ovarian cancer in this cohort was due to a Lynch syndrome (LS) germline pathogenic variant. Clinicians should consider germline testing for LS for all women with epithelial ovarian cancer.

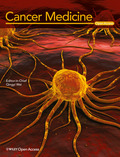

## 1 INTRODUCTION

Lynch syndrome (formerly known as hereditary non‐polyposis colorectal cancer; HNPCC) is a hereditary condition caused by a germline pathogenic or likely pathogenic variant in one of four DNA mismatch repair (MMR) genes: *MLH1*, *MSH2*, *MSH6*, and *PMS2*.^1^ It can also be caused by a deletion in *EPCAM*, which leads to hypermethylation of the *MSH2* promotor.^2^ Depending on the gene involved, women with Lynch syndrome (LS) have a lifetime ovarian cancer risk (including fallopian tube and primary peritoneal cancer) of 3%–17%.^3^ In an unselected population of women with ovarian cancer, <1% were found to carry a germline MMR gene pathogenic or likely pathogenic variant,^4^ however less is known about the incidence of LS in women with mismatch repair‐deficient (MMR‐D) ovarian cancer.

Multiple studies^5^, ^6^, ^7^, ^8^, ^9^, ^10^ have assessed the frequency of MMR‐D in ovarian cancers with 2%–13% shown to have MMR‐D, however, most did not report on subsequent germline testing. Bennett and colleagues,^8^ assessed clear cell ovarian cancer for pathological characteristics indicating MMR‐D and found 6% with MMR‐D. Four of the six patients with MMR‐D ovarian cancer underwent germline testing and all four patients were found to carry a germline mutation in the corresponding MMR gene. One of these cases is included in our cohort. Lantham and colleagues^9^ performed germline sequencing for 46 women with ovarian cancer identified to be MSI‐H and found none with LS. A recent study by Fraune and colleagues^10^ identified MMR‐D/MSI in 1.9% of screened ovarian cancers, with almost all tumors being endometrioid and suggested that testing for MMR‐D by IHC or PRC‐based analysis of microsatellite instability (MSI) should be routinely determined in endometrioid ovarian cancer. However, they did not evaluate germline LS in this cohort. The objective of this study was to clarify the prevalence of LS in women with MMR‐D ovarian cancer.

## 2 MATERIALS AND METHODS

Three hundred and eight cases of ovarian, fallopian tube, and primary peritoneal cancers underwent MMR staining by immunohistochemistry (IHC) by Stanford pathology between January 2012 and December 2019. This screening was unselected for age, stage, additional personal cancer history, or family history of cancer. From January 2012 to November 2016, MMR staining was performed for all epithelial ovarian cancers. From November 2016 to December 2019, MMR staining was performed for non‐serous, non‐mucinous epithelial ovarian cancers. This protocol change was made because MMR‐D was not being identified in serous and mucinous ovarian cancers.

Immunohistochemistry staining was performed on paraffin‐embedded tissue sections, using standard protocols, using monoclonal antisera reacting with MLH1 (clone G168‐728, BD PharMingen), MSH2 (clone FE11, Oncogene), MSH6 (clone 44, BD Transduction), and PMS2 (clone MRQ‐28, Cell Marque). Normal expression was defined as nuclear staining within tumor cells, using nuclei at the base of normal crypts (or infiltrating lymphocytes), as positive internal control. Tumors that showed a pattern of MLH1 and PMS2 protein loss had reflex *MLH1* promotor hypermethylation testing. Patients whose cancers showed MMR‐D without *MLH1* promotor hypermethylation (when this testing was indicated) were referred by their treating provider to cancer genetics either at Stanford (83% of patients) or an outside cancer genetics clinic (17% of patients).

Gene sequencing and variant classification were performed by one of several commercial clinical laboratories using an industry standard technique at the time of the testing. The laboratory was determined by the ordering clinician. Most testing utilized massively parallel sequencing technology with germline pathogenic or likely pathogenic variants (PV/LPV) confirmed with an orthogonal technology, such as Sanger sequencing or Array CGH. Single gene testing (e.g., *MLH1* only) was done by Sanger sequencing and multiplex ligation‐dependent probe amplification (MLPA) for deletion/duplication analysis. All multigene panel testing included at least sequencing and duplication/deletion analysis for *MLH1* NM_000249.3, *MSH2* NM_000251.2, *MSH6* NM_000179.2, and *PMS2* NM_000535.5 and duplication/deletion analysis for *EPCAM* NM_002354.2. All chosen laboratories included *PMS2* sequencing of exons 12–15 if *PMS2* testing was performed. For one case, germline testing was done in parallel with somatic tumor sequencing of the Lynch genes. Pedigree analysis was performed for all 12 cases suggestive of LS to determine if reported personal and family history met Amsterdam II criteria, including the proband's diagnosis of ovarian cancer as a criterion, as is common in clinical practice. Pathology re‐review of the 12 cases with MMR‐D without *MLH1* promotor hypermethylation was performed by authors TL and AKF in 2020 to confirm concordance of the pathological subtype among authors. Pathologists were not blinded to germline or MMR status at the time of the re‐review.

## 3 RESULTS

Sixteen of 308 ovarian/fallopian/primary peritoneal tumors (5.2%); (95% CI: 3.2%–8.3%) were MMR‐D. Cases with MMR‐D are summarized in Table 1; 14 cases were ovarian, one was a primary fallopian tube cancer, and one a primary peritoneal cancer. Of the 16 cases with MMR‐D, 11 were endometrioid (68.7%); (95% CI: 44.2%–86.1%), 4 cases were clear cell (25.0%) (95% CI: 9.71%–50.0%), and 1 case was a carcinosarcoma (6.25%) (95% CI: <0.01%–30.3%). Three cases were originally classified as either high‐grade or low‐grade serous, however upon pathology re‐review in 2020 two cases were reclassified as endometrioid and one reclassified as clear cell. The overall rate of MMR‐D of endometrioid tumors was 4% with the rate of MMR‐D of other histologies exceedingly low. The distribution of cases with MMR‐D was consistent over the 10‐year cohort.

**TABLE 1:** Patient demographics, tumor, and germline results

Patient number	Age at dx	Primary site	Pathology	MMR results	*MLH1* promotor hypermethylation (Yes 1)	LS Germline testing	PV/LPV (Yes = 1)	Gene involved	Predictive (Yes = 1)	Fam hx met Amsterdam II (Yes:1)	Additional information
1	55	Ovary	Endometrioid	Loss of MLH1 and PMS2	1	N/A	N/A	N/A	Epigenetic	N/A	
2	47	Ovary	Endometrioid	Loss of MLH1 and PMS2	1	N/A	N/A	N/A	Epigenetic	N/A	
3	50	Ovary	Endometrioid	Loss of MLH1 and PMS2	1	N/A	N/A	N/A	Epigenetic	N/A	
4	60	Ovary	Clear cell	Loss of MLH1 and PMS2	1	N/A	N/A	N/A	Epigenetic	N/A	
5	47	Ovary	Endometrioid	Loss of MSH2 and MSH6	N/A	*MSH2/MSH6*	0	Negative	0	N/A	
6	55	Ovary	Endometrioid	Loss of MSH2 and MSH6	N/A	Panel	0	Negative	0	N/A	
7	38	Ovary	Endometrioid	Loss of MSH2 and MSH6	N/A	Panel	1	*POLE*	0	N/A	*POLE* PV
8	47	Ovary	Endometrioid	Loss of MSH2 and MSH6	N/A	Paired tumor plus germline	0	Negative	0	N/A	Biallelic somatic *MSH2* PV/LPV
9	61	Ovary	Low‐grade serous (re‐review: endometrioid)	Loss of MSH2 and MSH6	N/A	*MSH2*	1	*MSH2*	1	1	
10	44	Ovary	Clear cell	Loss of MSH2 and MSH6	N/A	*MSH2*	1	*MSH2*	1	1	
11	51	Ovary	High‐grade serous (re‐review: clear cell)	Loss of MSH2 and MSH6	N/A	Panel	1	*MSH2*	1	0	
12	52	Primary peritoneal	Endometrioid	Loss of MSH2 and MSH6	N/A	Panel	1	*MSH2*	1	1	
13	52	Fallopian tube	High‐grade serous (re‐review: endometrioid)	Loss of MSH6	N/A	Panel	1	*MSH6*	1	0	
14	66	Ovary	Clear cell	Loss of MSH6	N/A	Panel	1	*MSH6*	1	1	
15	61	Ovary	Endometrioid	Loss of PMS2	N/A	*PMS2*	1	*PMS2*	1	0	
16	41	Ovary	Carcinosarcoma	Loss of PMS2	N/A	Panel	1	*PMS2*	1	1	

Abbreviations: dx, diagnosis; fam hx, family history; N/A, not applicable; PV/LPV, pathogenic variant/likely pathogenic variant.

Of the cases with MMR‐D, MSH2/MSH6 protein loss was detected in eight cases (50.0%); (95% CI: 28.0%–72.0%) and MLH1/PMS2 protein loss was detected in four cases (25.0%); (95% CI: 9.7%–50.0%). MSH6 protein loss was detected in two cases (12.5%); (95% CI: 2.2%–37.3%) and PMS2 protein loss was detected in two cases (12.5%); (95% CI: 2.2%–37.3%). All four cases with MLH1/PMS2 protein loss had *MLH1* promotor hypermethylation.

All 12 patients with ovarian cancer suggestive of LS (MMR‐D without *MLH1* promotor hypermethylation) were referred for pre‐test genetic counseling, underwent a consultation with a certified genetic counselor (CGC), and all opted to pursue germline genetic testing for LS. Eight patients (66.6%); (95% CI: 38.8%–86.5%) were found to have a germline PV/LPV confirming LS. All patients with a PV/LPV had a germline finding that corresponded to the original tumor staining; for example, an *MSH2* mutation identified in a patient whose tumor had MSH2/MSH6 protein loss on staining. The tumors of the four patients without LS all had MSH2/MSH6 protein loss and were endometrioid. Three of these four patients underwent multigene panel testing and one patient was identified to have a *POLE* PV, which may explain the staining pattern and is discussed further below. Another patient had confirmed *MSH2* biallelic somatic PV/LPV. The remaining two cases did not have an identifiable etiology to the initial staining pattern after germline testing and somatic tumor testing was not performed. Possible explanations include an unidentifiable germline PV/LPV, biallelic somatic PV/LPV, or another mechanism for MMR‐D. Of the eight patients with confirmed LS, three families (37.5%) did not meet Amsterdam II criteria.

LS germline testing was not systematically performed for this cohort at any timepoint. Germline testing would have been at the discretion of the ordering provider either within our Cancer Genetics clinic or with an outside provider. With the standard use of multigene panels starting in approximately 2013 in our clinic, many of the patients did choose multigene panel testing including LS. Of the cases for which LS germline testing was performed and we have access to germline results, no germline LS was identified in cases with proficient MMR.

## 4 DISCUSSION

More than 65% of patients with MMR‐D ovarian cancer in this cohort had a germline PV/LPV confirming LS. This may have significant implications for clinical practice and guideline development.

The results of this study contrast with those of a recent study that did not identify LS in patients with MSI‐H ovarian cancer.^9^ This discrepancy may be because of a different histologic subtype distribution of the two studies. A screening protocol change in this cohort to exclude mucinous and serous tumors after November 2016 enriched this cohort for non‐serous, non‐mucinous tumors. In this cohort of women with MMR‐D ovarian cancer, 69% of patients had endometrioid ovarian cancer, which are more often seen in LS‐associated ovarian cancer. In contrast, 67% of the cohort studied by Latham and colleagues^9^ were high‐grade serous ovarian cancer.

There is growing concern that Amsterdam criteria are insufficiently sensitive to identify patients with LS,^11^ especially for patients with ovarian cancer at increased chance of having LS,^12^ and the present results provide further evidence. Three out the eight patients with LS (37.5%) did not meet Amsterdam II criteria, so it is unclear if they would have been identified through typical referral patterns in which clinicians routinely use ovarian cancer as a criterion in Amsterdam II criteria. The three patients who did not meet Amsterdam criteria had PV/LPV in *MSH2* (1), *MSH6* (1), and *PMS2* (1).

There is controversy as to whether ovarian tumors should undergo universal MMR staining, as is standard for both colon and endometrial tumors. The findings from this study do not support universal MMR staining for all ovarian tumors given the small subset (5.2%) of ovarian cancer with MMR‐D. In contrast, 10%–15% of colon cancers and 20%–25% of endometrial cancers show MMR‐D.^9^ Fraune and colleagues^10^ suggest universal MMR staining specifically for endometrioid ovarian tumors, which would enrich for MMR‐D and may also allow for MSI‐H‐targeted therapy, such as with the immune checkpoint inhibitor Pembrolizumab. This is an important consideration for clinicians for treatment decisions, but universal MMR staining for ovarian cancer is likely not efficient for the purpose of identifying LS.

It is well known that there is a higher yield for actionable mutations when testing utilizes a multigene panel^13^, ^14^, ^15^ and multigene panel testing has largely replaced single gene or single syndrome testing.^16^ The National Comprehensive Cancer Network (NCCN) guidelines recommend germline testing of high‐penetrance breast and/or ovarian cancer susceptibility genes, which they identify as *BRCA1*, *BRCA2*, *CDH1*, *PALB2*, *PTEN*, and *TP53* for all women with epithelial ovarian cancer, regardless of pathology.^17^ However, NCCN does not currently make a specific recommendation for LS testing for women with ovarian cancer, thus it is at the clinician's discretion whether LS testing is offered or included along with these high‐penetrant breast/ovarian cancer genes. Some clinicians may not be aware of the germline associations of specific ovarian cancer histologies and may choose to offer *BRCA1* and *BRCA2* testing only, even in the context of non‐serous, non‐mucinous ovarian cancer. This decreases the sensitivity of identifying LS and limits the opportunity for cancer prevention and early detection for other LS‐associated tumors for both the patient and her relatives. If the objective is to identify women with LS, the findings from the study suggest going directly to multigene germline testing in these cases.

Unfortunately, recent data have shown a general under‐utilization of germline testing for women with ovarian cancer, with fewer than one‐third of women undergoing some form of germline testing in the two states studied.^18^ The overall prevalence of germline LS for women with non‐serous, non‐mucinous ovarian cancer in this cohort for whom results were available was 8 out of 96 cases (8.3%). No cases of germline LS were identified for the women with serous, mucinous, or unknown histology for whom LS germline testing was performed and results were available. Given these potential barriers, multigene panel testing that includes LS testing for patients with ovarian cancer is more cost and time efficient than initial MMR staining and separate high‐penetrance breast and/or ovarian susceptibility germline testing, especially for women with non‐serous, non‐mucinous ovarian cancer.

Multigene panel testing in this cohort identified a non‐Lynch PV in *POLE*. This patient had a personal and family history of previously unexplained colonic adenomatous polyposis. Previous patients with a germline *POLE* mutation have presented with MSI‐H colon tumors.^19^, ^20^ There has been at least one case of ovarian cancer in a family with polymerase proofreading‐associated polyposis (PPAP) caused by a germline *POLE* PV, but the MSI status of the ovarian tumor was not known or reported.^21^ Given the molecular mechanism of PPAP in carcinogenesis and the characteristics of the MSI‐H colon cancers previous reported, the identified POLE PV may be the etiology of this identified ovarian tumor with MMR‐D. This needs additional clarification. Identification of the *POLE* PV was a clear etiology of the previously unexplained familial polyposis. This complexity with germline results is an important discussion point for patient informed consent and shared decision making about which genes are included on multigene panel testing. Current guidelines recommend a health professional with expertise and experience in cancer genetics be involved in both pre‐test and post‐test counseling whenever possible.^17^

These results emphasize the importance of germline LS genetic testing for women with MMR‐D ovarian cancer in the setting of initial tumor screening. It also suggests that all women with non‐serous, non‐mucinous epithelial ovarian cancer should be offered LS germline testing even without initial tumor screening, given the higher yield for LS with these histologies.

## 5 IRB

IRB approval for this study under Stanford protocol ID 12234.

## CONFLICT OF INTEREST

Authors RH, KK, KC, AKF, and TL declare no conflict of interest. Authors AWK and JMF declare Myriad research funding for an unrelated project from 2017–2019.

## AUTHOR CONTRIBUTIONS

RH and KK conceptualized the study and contributed to study design. AKF and TL did analysis of immunohistochemical staining and histology of all cases with mismatch repair deficiency and TL supervised data curation. KC did initial data curation and abstract writing. RH did additional data curation, formal analysis, abstract writing, and manuscript original draft writing. AK and JMF contributed supervision and critical revision. All authors reviewed, edited, and approved the final manuscript.

## Data Availability

The data that support the findings of this study are available on request from the corresponding author. The data are not publicly available due to privacy or ethical restrictions.
